# Expanding horizons of cancer immunotherapy: hopes and hurdles

**DOI:** 10.3389/fonc.2025.1511560

**Published:** 2025-04-25

**Authors:** Priyanka Vijay Sonar, Anuj Kumar Singh, Sravan Mandadi, Nilesh Kumar Sharma

**Affiliations:** ^1^ Cancer and Translational Research Lab, Dr. D.Y. Patil Biotechnology & Bioinformatics Institute, Dr. D.Y. Patil Vidyapeeth, Pune, Maharashtra, India; ^2^ Ichnos Glenmark Innovation, Glenmark Pharmaceuticals Limited, Navi Mumbai, Maharashtra, India

**Keywords:** cancer drug resistance, gut microbiome, immune checkpoint inhibitors, immunotherapy, neoplasms

## Abstract

**Background:**

Tumor displays various forms of tumor heterogeneity including immune heterogeneity that allow cancer cells to survive during conventional anticancer drug interventions. Thus, there is a strong rationale for overcoming anticancer drug resistance by employing the components of immune cells. Using the immune system to target tumor cells has revolutionized treatment. Recently, significant progress has been achieved at preclinical and clinical levels to benefit cancer patients.

**Approach:**

A review of literature from the past ten years across PubMed, Scopus, and Web of Science focused on immunotherapy strategies. These include immune checkpoint inhibitors (ICIs), tumor-infiltrating lymphocyte therapy, antibody-drug conjugates (ADCs), cancer vaccines, CAR T-cell therapy, and the role of the gut microbiome.

**Conclusion:**

While immunotherapy outcomes have improved, particularly for tumor types such as melanoma and non-small cell lung cancer (NSCLC), challenges persist regarding predictive biomarker identification and better management. Ongoing research on modifiers of immune function like gut microbiome-derived metabolites, next-generation ADCs, and new classes of biologics is warranted. Overall, continued investigation toward optimizing synergistic immunotherapeutic combinations through strategic drug delivery systems is imperative for preclinical and clinical success in cancer patients.

## 1 Introduction

For a long time, cancer has presented a significant obstacle to medical science, and conventional treatment methods frequently fail to offer long-term benefits. Recently, cancer immunotherapy has emerged as a breakthrough approach to treating various malignancies (1–4). Cancer immunotherapy, which uses the body’s immune system to combat cancer, has significantly improved patient outcomes. Cancer immunotherapy includes living medicines such as chimeric antigen receptor (CAR)-T cells, tumor-infiltrating lymphocytes (TILs), and non-living drugs such as monoclonal antibodies, and immune checkpoint inhibitors (ICIs). Cancer immunotherapy is an essential clinical strategy to improve anti-tumor immune responses (5–9). Nevertheless, cancers may become resistant to immune monitoring, which could result in low response rates and ineffective treatment (10–14). Research on the role of gut microbiome in cancer immunotherapy is encouraging. Certain gut bacteria may improve the body’s response to ICIs, potentially converting non-responders into responders (15–18).

Tumor cells altered signaling pathways, genetic modifications, and the patient’s microbial signature are the main causes of this resistance, which makes them less receptive to immunotherapeutic treatments (15–21). Tumors are capable of creating an immunosuppressive environment by releasing molecules and attracting cells that block immune cell infiltration and function (9, 22–31).

Various types of immunotherapy strategies have been developed, each with distinct mechanisms and targets (30–41). Checkpoint inhibitors are drugs that release the brakes on the immune system, allowing it to recognize and attack cancer cells more effectively. By blocking certain proteins, checkpoint inhibitors help unleash the immune system’s full potential. In CAR-T cell treatment, a patient’s T cells are engineered to express a particular receptor capable of identifying cancer cells (42–52). These systems are designed to restore or enhance immune cell activity and amplify immune responses. The modified T cells are then reintroduced into the patient, where they become more effective at recognizing and eliminating cancer cells. Monoclonal antibodies, which are lab-produced molecules, can specifically identify and bind to targets on cancer cells. By doing so, they can either directly destroy cancer cells or stimulate an immune system response against them (53–63).

The gut microbiome, comprising the diverse community of microorganisms residing in the gastrointestinal tract, has garnered recognition as a crucial factor in immunotherapy (64–70). Researchers have suggested that the gut microbiome holds potential as both biomarkers and targets for manipulation to predict and augment the effectiveness of antitumor immunotherapy across various cancer types (71–77). Efforts to modify the gut microbiome using probiotics, prebiotics, antibiotics, or fecal microbiota transplantation (FMT) are being explored to improve immunotherapy outcomes (75–77).

Immunotherapy has altered the way that cancer is treated, but there are still some issues. Multidisciplinary cooperation, ongoing research, and creative thinking are needed to overcome these obstacles. This paper explores the potential of immunotherapy in cancer treatment, focusing on overcoming resistance, identifying predictive biomarkers, managing immune-related adverse events (irAEs), improving affordability, optimizing combination therapies, enhancing smart drug delivery systems (SDDSs), and leveraging the gut microbiome (GM).

## 2 Tumor immune heterogeneity

Tumor cells acquire several hallmark capabilities that enable their malignant growth and spread. These include sustaining proliferative signaling, evading growth suppressors, resisting cell death, enabling replicative immortality, inducing angiogenesis, and activating invasion and metastasis (15–17). Tumors also exhibit genome instability and inflammation, which facilitate tumor progression. Most critically, tumors evolve mechanisms to avoid immune destruction, known as immune evasion. This capability allows tumors to suppress, inactivate, or avoid detection by the immune system (18–21). Immunosuppressive cells, inhibitory receptors, cytokines, and disrupted antigen presentation weaken anti-tumor immune responses. Overcoming these barriers is crucial for successful immunotherapy.

A key feature underlying immune evasion is marked by heterogeneity within the tumor and its microenvironment. This heterogeneity spans multiple dimensions at genetic, epigenetic, phenotypic, functional, and microenvironmental levels. Intra-tumoral immune heterogeneity involves spatial, temporal, and genomic variations in the abundance, composition, functional orientation, and organization of immune cells (19–21). Tumor heterogeneity allows resistant subclones to grow, helping the tumor evade treatment. These differences determine whether a tumor is inflamed with immune cells or suppressed by regulatory components. Monitoring immune profiles over time and space is crucial to understanding resistance. Advanced profiling of tumor-infiltrating lymphocytes and myeloid cells has identified immune subtypes linked to different responses. Understanding the causes and patterns of heterogeneity can help overcome treatment failure (9, 22–24).

The immune landscape of tumors critically impacts their susceptibility to immunotherapies like immune checkpoint blockade. However, the development of resistance remains a key limitation. Tumors exploit numerous tactics to evade immune attack and blunt immunotherapeutic efficacy (9, 15–24). These include the loss of immunogenic antigens, defects in antigen presentation, increased expression of alternative immune checkpoints, recruitment of immunosuppressive cells, and the release of inhibitory cytokines. Therapies initially effective at unleashing anti-tumor immunity eventually confront resistant subclones or a recalibrated microenvironment (9, 19–24). Even inflamed tumors with high T-cell infiltration can acquire resistance. Tracking the evolution of the immune contexture and escaping subclones is essential to therapeutic durability. Strategies to overcome resistance include disrupting suppressive networks, boosting T cell function, eliminating immunosuppressive cells, converting them into immune-activating cells, and targeting non-immunogenic tumor niches (9, 24). Ultimately, deciphering and redirecting immune heterogeneity in space and time provides a path to more effective and sustained immunotherapy responses.

## 3 Cancer immunotherapy

Cancer immunotherapy, also referred to as biologic therapy, is a ground-breaking treatment modality that aims to enhance the body’s immune system to recognize and destroy cancer cells (9, 21–24). Unlike chemotherapy, radiation, or targeted therapy, which directly attack cancer cells, immunotherapy boosts the immune system to fight cancer (9, 25–27). The major plus point of immunotherapy lies in the fact that it damages only the cancerous cells and the healthy cells remain minimally unaffected. This reduces the side effects of cancer treatment as well it puts patients’ lives at a lesser risk of death by cancer (9, 25–31).

Immunotherapy has revolutionized cancer treatment. The idea of using the immune system to fight disease dates back centuries. In the late 19th century, William Coley noticed that some cancer patients went into remission after bacterial infections. This led to the foundation of immunotherapy by stimulating the immune response against cancer (9). Researchers in the early 20th century, including Paul Ehrlich and Emil von Behring, explored immune stimulation by developing serums and antibodies to combat diseases like diphtheria and tetanus. These studies demonstrated the potential of immune-based interventions (25).

**Figure 1:**
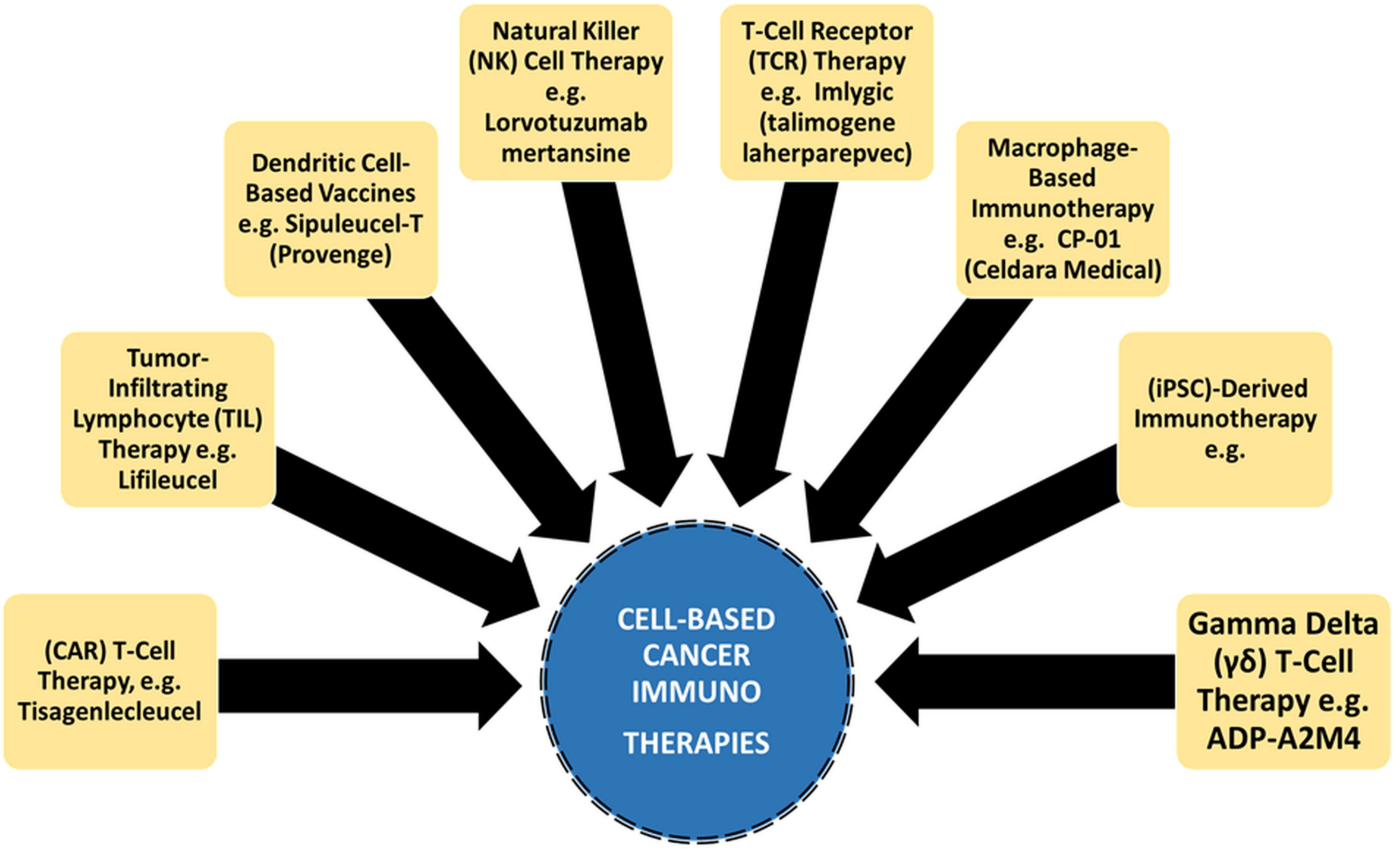
Cancer immunotherapies are attributed with targeted and enhanced anti-tumor response over traditional cancer therapy.

The field of tumor immunology emerged in the mid-20^th^ century when Lewis Thomas and others began investigating the interaction between the immune system and cancer cells. They observed the presence of immune cells within tumors and recognized the potential for immune responses against cancer. In the 1960s, interferons were discovered as natural proteins with potent antiviral and antitumor properties. Interferon therapy represented one of the earliest attempts at immunotherapy for cancer treatment (26). In 1975, Georges Köhler and César Milstein pioneered the hybridoma technique, enabling the production of large quantities of specific mAbs (27, 28). Rituximab, the first FDA-approved mAb for treatment, marked a significant discovery. James Allison and Tasuku Honjo’s discoveries of cytotoxic T-lymphocyte-associated protein 4 (CTLA-4) and programmed cell death protein 1 (PD-1), respectively, opened new avenues for cancer immunotherapy (29, 30). Immune checkpoint inhibitors, such as PD-1 inhibitors (nivolumab, pembrolizumab, cemiplimab), CTLA-4 inhibitor (ipilimumab), and PD-L1 inhibitors (atezolizumab, durvalumab, avelumab), have transformed cancer treatment by helping the immune system recognize and attack cancer cells (9, 15–24). Adoptive cell transfer (ACT) therapy modifies immune cells-specifically, T cells-in patients to improve their capacity to combat cancer. Carl June and associates invented the chimeric antigen receptor (CAR) T-cell therapy, which has shown promise in the treatment of several blood malignancies (31). A summarized flow model on the rationale of cancer immunotherapies is presented (**Figure 1**).

## 4 Types of immunotherapies

Immunotherapy includes different types, each targeting specific aspects of the immune response to cancer. These treatments offer new hope for lasting responses, better survival rates, and improved quality of life for cancer patients. This section explores their mechanisms and clinical impact.

### 4.1 Immune checkpoint inhibitors

Immune checkpoint proteins are known to suppress the immune system through various pathways that can prevent harm to healthy tissues. However, cancer cells exploit immune checkpoint proteins such as CTLA4 and PD1 to evade immune attack. Encouraging preclinical and clinical developments have been reported on immune checkpoint inhibitors (ICIs) that can block these proteins and in turn allowing immune cells to recognize and attack cancer cells (17, 32–41). Two key checkpoint proteins, CTLA-4 and PD-1, play crucial roles in regulating the function of T cells. CTLA-4 dampens T-cell activation by engaging with its ligands, while PD-1 modulates T-cell activity in peripheral tissues through its interaction with PD-L1 and PD-L2 (32). ICIs have displayed promise as therapeutic interventions for cancer by reversing the immune system’s suppressive effects on tumor immunity (33, 34).

Early investigations showcased the effectiveness of anti-CTLA-4 therapy in mouse models of tumors, whereas blocking PD-1 or PD-L1 bolstered anti-tumor immune responses. Consequently, antibodies targeting CTLA-4, PD-1, and PD-L1 were developed to hinder their interaction with respective ligands. Clinical trials involving antibodies such as ipilimumab and nivolumab, aimed at CTLA-4 and PD-1/PD-L1 respectively, demonstrated lasting responses in melanoma and other cancers. However, they were also linked to severe irAEs like colitis, dermatitis, thyroiditis, pneumonitis, and hepatitis (35). Efforts have been made to explore combination therapies targeting multiple immune checkpoints, leading to increased response rates albeit with a higher occurrence of severe irAEs. Besides the CTLA-4 and PD-1/PD-L1 pathways, other inhibitory receptors like LAG-3, TIM-3, and TIGIT have been targeted in combination with anti-PD-1 therapy. Nevertheless, the potential severity and lethality of ICI-associated toxicities have become apparent. Fatalities, occurring sporadically and early during treatment, exhibit different patterns of organ involvement between anti-CTLA-4 and anti-PD-1/PD-L1 therapies (36, 37). Colitis is more frequently associated with anti-CTLA-4 treatment, while pneumonitis, hepatitis, and neurotoxicity are more observed with anti-PD-1/PD-L1 treatment.

ICIs have revolutionized cancer treatment, including malignancies previously deemed untreatable (38). These agents can incite inflammation and tissue damage in various organs. Myocarditis, a rare yet severe toxicity associated with ICIs, has garnered attention due to its often swift and fatal progression. The underlying mechanisms and specific antigens that trigger ICI-associated myocarditis remain poorly understood (39–41). Deaths related to combination therapy frequently stem from colitis or myocarditis, with the latter exhibiting the highest fatality rate among irAEs (40-50%). It is imperative to develop strategies that effectively manage ICI-associated toxicities without compromising their anticancer efficacy. While ICIs have shown encouraging responses in some cancer patients, however, develop resistance over time is a concern driven by various alterations in the tumor microenvironment.

**Figure 2:**
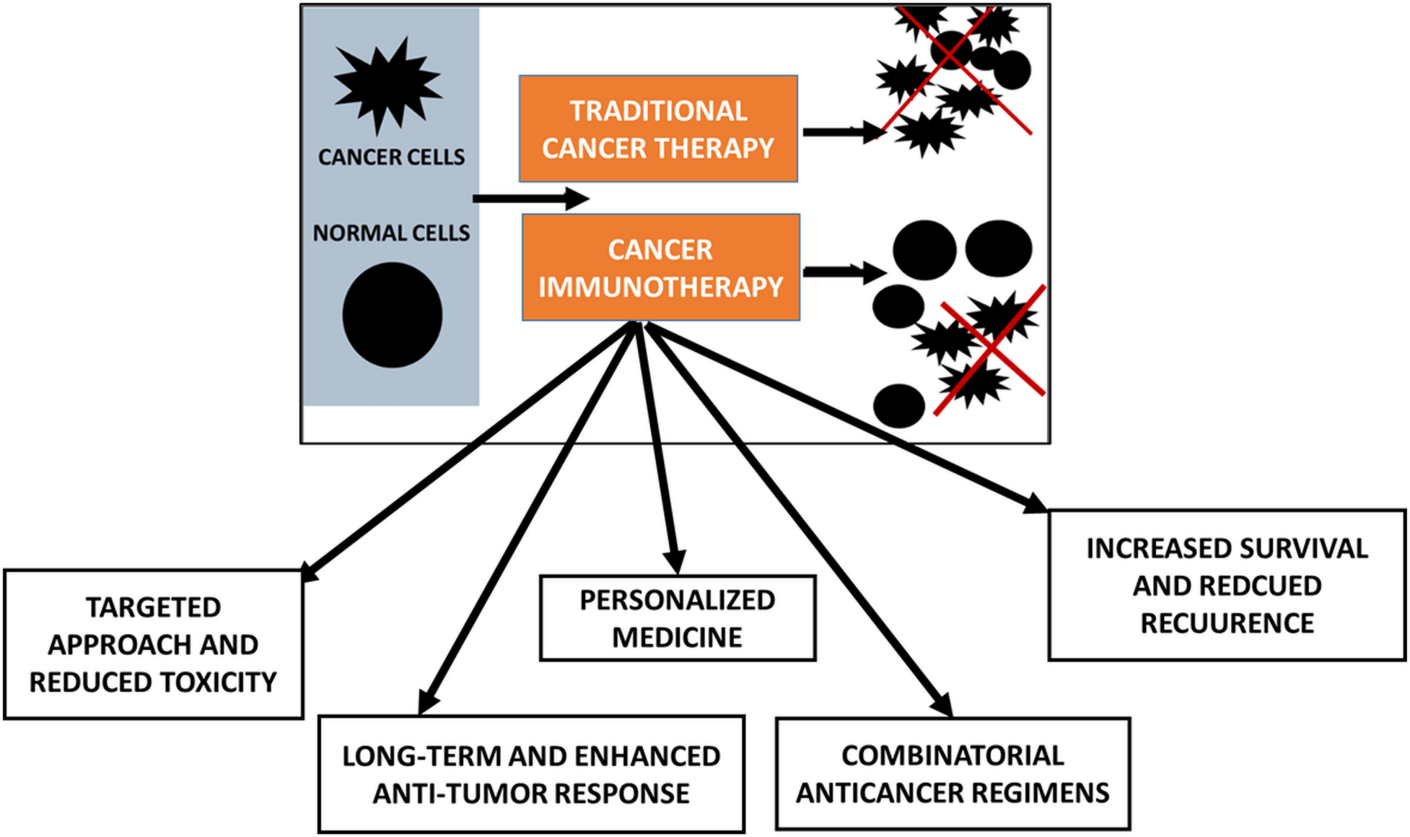
Types of immune checkpoint inhibitors (ICIs) with clinically approved drugs.

Primarily, tumors fail to respond from the start due to poor immune cell infiltration or lack of antigens for T-cell recognition (38–40). At the same time, acquired forms of resistance due to mutations reduce antigen presentation or increase immune checkpoint molecules. Also, immune-mediated resistance is observed with the decline of T-cell functions and the emergence of immunosuppressive cells. In the context of the pro-tumor microenvironment, various non-cellular components such as immunosuppressive molecules or cellular components in the stromal cells that hinder the immune response (39, 40). A summary of the various classes of cancer immunotherapies with suitable examples of clinical drugs is provided (**Figure 2**).

### 4.2 Chimeric antigen receptor T-cell therapy

Beyond antibody-based techniques, the use of autologous T cells modified by gene transfer to express receptors specific to chemicals found on malignant cells offers promise in cancer therapies (42, 43). This technique entails inserting genes encoding antigen receptors into the patient’s T cells, which are then grown *in vitro* to produce memory and effector lymphocytes capable of vigorous proliferation in the body, ultimately exerting potent anti-tumor effects (44, 45). Using chimeric antigen receptors (CARs) to genetically reroute and reprogram T cells is a noteworthy method of bypassing cancer tolerance. Synthetic receptors enable precise targeting of surface antigens without requiring Major Histocompatibility Complex (MHC) molecules. They combine T-cell functions with the antigen-recognition ability of antibodies (46). Liquid cancers are better suited for this kind of treatment.

More than thirty years ago, the first synthetic immunoglobulin/TCR chimeric molecule with antibody-like specificity was expressed, sparking the start of the development of CARs. Later research showed that T cells may be activated by a CAR made up of CD8 and the CD3ζ chain without the need for their native TCR (47). CARs typically comprise an external domain that identifies tumor antigens and one or more intracellular signaling regions that cause T-cell activation (48, 49). A major advantage of CAR-based immunotherapy is that scFv (Single-chain variable fragments) from antibodies have a much higher affinity than TCRs. CAR T cells can directly target intact surface proteins without needing antigen processing or MHC presentation, reducing the risk of tumor escape (50–52). Moreover, CARs make it possible to recognize antigens that TCRs may miss or find difficult to recognize, such as glycolipids, abnormally glycosylated proteins, and conformational epitopes.

### 4.3 Tumor-infiltrating lymphocyte therapy

As a type of adoptive cellular therapy, TIL therapy comprises harvesting lymphocytes that have penetrated tumors, growing and amplifying them *in vitro*, and then infusing the cells back into patients to treat them (53). TIL therapy offers unique advantages over other adoptive cell therapies for solid tumors. It has better tumor-targeting ability, lower off-target toxicity, and diverse T-cell receptor (TCR) clonality (54). TIL therapy was developed by extracting TIL from several mouse tumor models for the use of TIL therapy in human advanced cancers (55).

TIL treatment was tried clinically for the first time, and in patients with metastatic melanoma, it had an objective response rate of 60%. However, the features of solid tumors present serious obstacles to the creation of successful adoptive cellular therapies (56). Solid tumors are highly diverse, making it hard to find a universal target, unlike blood cancers with clear markers. Targeting a single antigen can lead to resistant clones or antigen loss. Even with many adoptively transferred T cells, solid tumors often show limited infiltration (57). The immunosuppressive tumor microenvironment (TME) contains regulatory T cells (Tregs), myeloid-derived suppressor cells (MDSCs), and tumor-associated macrophages (TAMs). These cells weaken T-cell function by increasing immune-inhibitory molecules, cytokines, and metabolites while reducing co-stimulatory molecules (58, 59). Compared to other adoptive cellular therapies like CAR-T and TCR-T cell therapies, tumor heterogeneity is more efficiently addressed by TIL, which is made up of T cells with various TCR clones that can recognize a variety of tumor antigens.

Notably, in solid tumors with high mutation loading, like melanoma, TIL has proven to be more clinically effective than CAR-T treatment. TIL cells are mostly made up of effector memory T (TEM) cells that express chemokine receptors including CCR5 and CXCR3, having been triggered by tumor antigens in the body (60). After being transferred into patients, TIL can readily move to antigenically different tissues, such as malignancies, because of their receptors. Because TCRs were negatively selected during the early stages of T cell immunity formation, TIL treatment has therefore demonstrated minimal off-target damage (61). On the other hand, if tailored tumor-targeting molecules show cross-reactivity with antigens found in normal tissues, such as single-chain variable segments in CAR-T or affinity-enhanced TCRs in TCR-T cell treatments, it could be harmful. In the treatment of melanoma, this strategy has demonstrated encouraging outcomes, with some patients seeing significant response rates (62, 63). Optimizing TIL treatment and expanding its efficacy to cover additional cancer types are ongoing endeavors. A summary on the comparison between different adoptive cellular therapies, clarifying their advantages and limitations is presented (**Table 1**).

**Table 1:** A summary on the comparison between different adoptive cellular therapies, clarifying their effectiveness, advantages and limitations are presented.

Feature	TIL Therapy	CAR-T Therapy	TCR-T Therapy
**Target Type**	Tumor-infiltrating lymphocytes (polyclonal, recognizing multiple tumor antigens)	Single-chain variable fragment (scFv) targeting specific surface antigens	TCR engineered to recognize intracellular antigens via MHC
**Tumor Targeting**	Broad, due to TCR clonality diversity	Narrow, depends on selected antigen	Broader than CAR-T but still limited to MHC-restricted antigens
**Effectiveness in Solid Tumors**	High, especially in tumors with high mutation load (e.g., melanoma)	Limited due to poor tumor infiltration	More effective than CAR-T but still affected by antigen heterogeneity
**Antigen Recognition**	Recognizes both intracellular and surface antigens	Only targets surface antigens	Recognizes intracellular antigens via MHC presentation
**Tumor Infiltration**	High due to natural homing ability (CCR5, CXCR3 expression)	Poor infiltration in solid tumors	Moderate infiltration
**Off-Target Toxicity**	Low, as TCRs undergo natural negative selection	Higher risk if target antigen is expressed in normal tissues	Moderate, as affinity-enhanced TCRs may cross-react
**Challenges**	Tumor heterogeneity, immunosuppressive TME	Antigen loss, poor infiltration, TME barriers	Requires MHC compatibility, risk of cross-reactivity

TIL Therapy: Tumor-Infiltrating Lymphocyte Therapy; CAR-T Therapy: Chimeric Antigen Receptor T-cell Therapy; TCR-T Therapy: T-cell Receptor Therapy.

Target Type, refers to the specificity of the T cell or engineered receptor used to recognize tumor antigens; Tumor Targeting, describes the range and diversity of tumor antigens that the therapy can effectively recognize and target; Effectiveness in Solid Tumors, indicates the therapeutic efficacy of the approach in treating solid malignancies; Antigen Recognition, specifies whether the therapy detects extracellular (surface) or intracellular antigens via MHC presentation; Tumor Infiltration, measures the ability of therapeutic T cells to migrate into and penetrate tumor tissue; Off-Target Toxicity, assesses the likelihood of unintended cytotoxicity against healthy tissues expressing similar antigens; Challenges, highlights key biological and technical barriers limiting the therapy's effectiveness or applicability.

### 4.4 Antibody-drug conjugate

The notion of antibody-drug conjugate (ADC) is a revolutionary one that combines the powerful efficacy of cytotoxic medications with the targeting capability of monoclonal antibodies (mAbs) (64–73, 78–80). ADCs provide accurate targeting and concurrently powerful therapeutic effects by conjugating mAbs to cytotoxic payloads using specific chemical linkers (64). Attaching a large hydrophilic antibody improves the therapeutic index by preventing the toxic payload from entering cells without the target antigen. In 2000, the FDA approved Mylotarg^®^ (gemtuzumab ozogamicin), the first ADC drug for acute myeloid leukemia (AML), marking the beginning of the ADC era in targeted cancer therapy (65, 66).

The three parts of an ADC are the chemical linker, the cytotoxic payload, and the antibody. The antibody has minimal immunogenicity and a long plasma half-life, and it selectively binds to the target antigen produced on tumor cells, facilitating effective internalization (67). The linker, which connects the antibody to the cytotoxic drug, determines ADC stability and payload release. ADCs bind to tumor cells, get internalized, and release their toxic payload into lysosomes. This triggers cell death or apoptosis through mechanisms like targeting microtubules or DNA (68). Because ADCs have a low oral bioavailability and are easily broken down by digestive enzymes, they are usually given intravenously. ADCs are anticipated to revolutionize cancer therapy by offering a focused and effective therapeutic strategy, maybe taking the place of traditional chemotherapies, thanks to their growing targets and indications. ADCs are a potentially beneficial tactic in the fight against cancer due to their accurate targeting, efficient killing of cancer cells, improvement of the therapeutic window, and reduction of off-target side effects. ADCs’ lethal effects are further amplified by the bystander effect and possible influence on the tumor microenvironment (69, 70).

As of present, the European Medicines Agency (EMA) has approved four antibody-drug conjugates (ADCs), while the FDA has given regulatory approval for nine ADCs in the US (78, 79). Kadcyla^®^ is an ADC approved by the FDA in 2013 and used to treat metastatic HER2-positive breast cancer. It combines the cytotoxic payload DM1 (Derivative of Maytansine 1) with the HER2-targeting monoclonal antibody trastuzumab (80). In 2011, the FDA approved Adcetris^®^ for treating systemic anaplastic large-cell lymphoma and Hodgkin lymphoma. This ADC consists of the CD30-targeting antibody brentuximab and the antimitotic drug MMAE. Its strong clinical results have provided patients with more treatment options (71, 72). The FDA approved Padcev^®^ in 2019 to treat metastatic urothelial carcinoma. It comprises MMAE, a chemical that disrupts microtubules, and enfortumab, a monoclonal antibody that targets nectin-4. Patients who have previously had immune checkpoint inhibitors and platinum-based chemotherapy have shown improvement with Padcev^®^ (73). In 2019, the FDA approved Enhertu^®^ to treat metastatic HER2-positive breast cancer. In 2019, the FDA approved Enhertu^®^ for metastatic HER2-positive breast cancer. This ADC combines trastuzumab, a HER2-targeting antibody, with deruxtecan, a topoisomerase I inhibitor. It has shown strong clinical responses, especially in patients resistant to previous HER2 therapies (74). Patients with restricted therapy options now have treatment options thanks to these ADCs, which have shown extraordinary success in treating a variety of cancers and having safety profiles (including colorectal, breast, and lymphoma cancer). We may expect the creation of new ADCs and the extension of their uses in the fight against cancer as long as research in this area continues.

### 4.5 Cancer vaccines

The advancement of cancer immunotherapy has given rise to several instruments and methods for thwarting the immune-evading processes employed by cancer cells. These resources include immunocompetent cells such as T and dendritic cells, as well as antibodies, peptides, proteins, and nucleic acids (75–77, 81–86). Based on their structure and substance, cancer vaccines developed with these methods can be divided into three primary classes. Cell vaccines fall within the first group; they are a type of immunotherapy that employs immune or tumor cells (77). Tumor cell vaccines use modified tumor cells to trigger an immune response against cancer-specific antigens. In contrast, immune cell vaccines enhance and modify immune cells, like T and dendritic cells, to boost their ability to recognize and attack cancer (81). Provenge (sipuleucel-T; Dendreon Corporation) received FDA approval in 2010 as a prostate cancer vaccine. Since then, it has attracted a lot of interest in the field of autologous immune cell-based immunotherapy (82). Vaccines based on particular proteins or peptides derived from tumor-associated antigens fall into the second category. The ability of these antigens to trigger an immune response against cancer cells is the basis for their selection. The immune system may identify and target cancer cells that express the matching antigens by delivering these protein/peptide vaccines, which trigger an anti-tumor immune response (83). An immunostimulatory adjuvant-based strategy has been developed to improve the immunogenicity and effectiveness of peptide vaccines.

Nucleic acid vaccines, which include DNA, RNA, and viral vectors used to transfer genetic material encoding tumor-associated antigens, make up the third category. The goal of this strategy is to ingest the genetic instructions necessary for the patient’s cells to produce these antigens directly (84–86). By doing this, the antigens can be produced internally by the patient’s cells, which will then mount an immunological defense against cancer cells that express those antigens.

**Figure 3:**
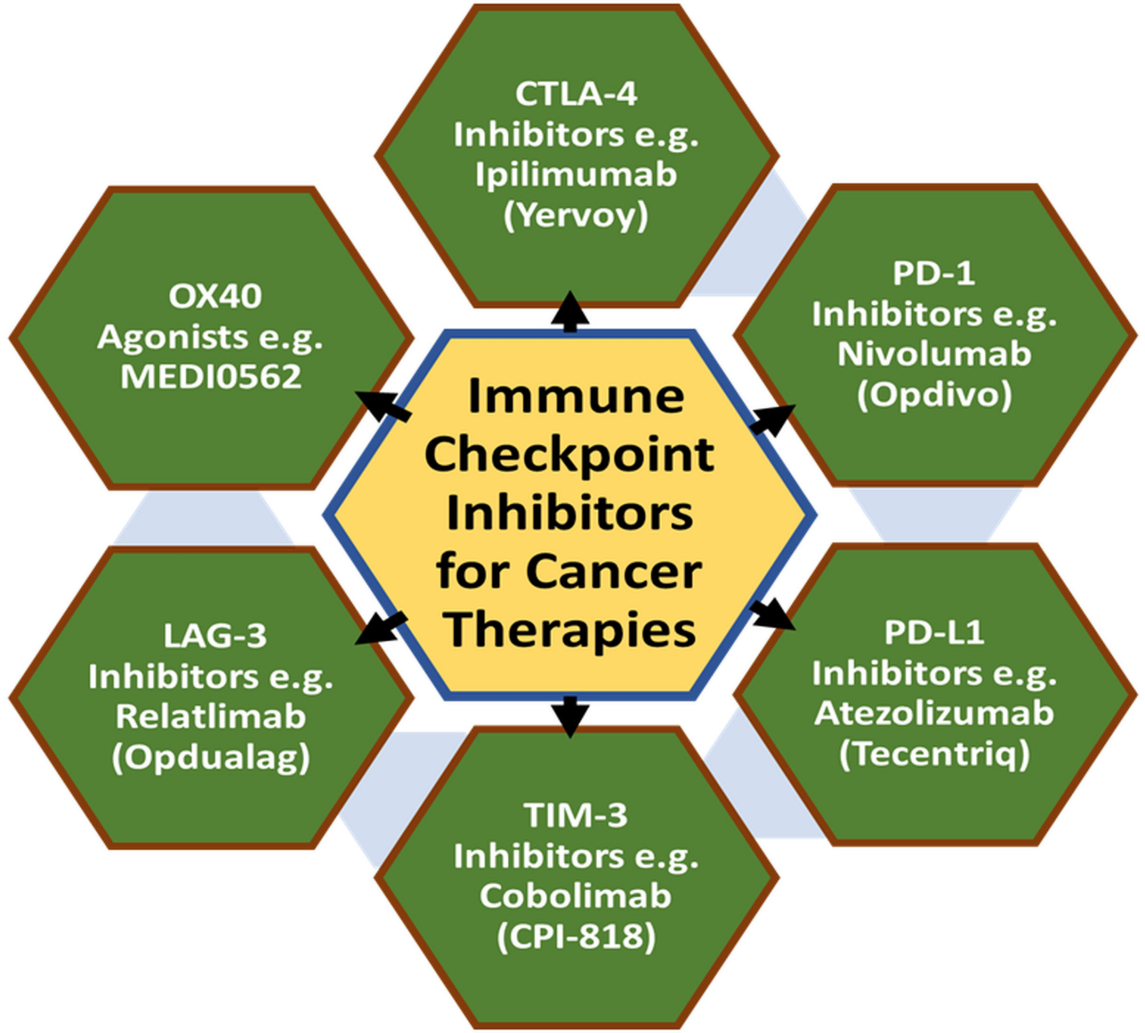
Types of cell-based cancer immunotherapies with suitable examples of clinical drugs.

A summarized flow model is depicted to highlight the cell-based cancer immunotherapies with suitable clinically approved anticancer drugs (**Figure 3**). Also, a tabular summary of various cancer immunotherapy approaches with comparisons of mechanisms, efficacy, and advantages is given (**Table 2**).

**Table 2:** Cancer Immunotherapy Approaches: Comparisons on mechanisms, efficacy, and advantages.

Immunotherapy Approach	Mechanism of Action	Clinical Efficacy	Key Advantages	References
Immune Checkpoint Inhibitors (ICIs)	Block negative regulators (e.g., PD-1, CTLA-4) to enhance T-cell response	20-50% ORR in various cancers (e.g., melanoma, lung, kidney)	Improved survival, durable responses	(32–34)
CAR-T Cell Therapy	Genetically modified T-cells recognize and attack cancer cells	50-90% ORR in B-cell malignancies (e.g., ALL, DLBCL)	High response rates, potential for cure	(87–93)
Cancer Vaccines	Stimulate immune response against tumor antigens	10-30% ORR in various cancers (e.g., prostate, lung)	Safe, potential for combination therapies	(4, 75–77, 81, 94–101)
Adoptive T-Cell Therapy	Infuse expanded, tumor-specific T-cells	50-70% ORR in melanoma and other cancers	Personalized, potential for long-term responses	(102–105)
Monoclonal Antibodies	Target tumor-associated antigens (e.g., HER2, EGFR)	10-50% ORR in various cancers	Well-established, targeted therapy	(98, 106)
Oncolytic Viruses	Selectively infect and kill cancer cells, stimulate immunity	10-30% ORR in various cancers	Novel approach, potential for combination therapies	(13)
Tumor-agnostic Therapies	Target shared tumor antigens (e.g., TMB, MSI-H)	20-50% ORR in various cancers	Potential for broad applicability	(91)
Combination Immunotherapies	Combine ICIs, CAR-T, vaccines, or other approaches	Enhanced efficacy, potential for synergistic effects	Promising clinical trials, improved outcomes	(107–118)

ORR, Overall Response Rate; ALL, Acute Lymphoblastic Leukemia; DLBCL, Diffuse Large B-Cell Lymphoma; TMB, Tumor Mutational Burden; MSI-H, Microsatellite Instability-High.

## 5 Preclinical and clinical trial advances

Immunotherapy has witnessed remarkable success in the treatment of various cancers. Checkpoint inhibitors have revolutionized the management of melanoma, lung cancer, and renal cell carcinoma. Clinical trials have shown that patients treated with these inhibitors experience improved survival rates and prolonged progression-free periods compared to conventional treatments (87–93, 107, 119–124). CAR-T cell therapy has shown outstanding success in blood cancers like acute lymphoblastic leukemia and diffuse large B-cell lymphoma. It has led to complete remission in patients who did not respond to traditional treatments (108–117).

Polymerase Proofreading-Associated Polyposis (PPAP) or POLE-mutated and Microsatellite Instability (MSI) endometrial cancers have high tumor-infiltrating lymphocytes and neoantigens. This suggests they may respond well to immunotherapy, especially PD-1/PD-L1 checkpoint inhibitors (88–93, 122–124). In a phase 2, pembrolizumab, an anti-PD-1 antibody, was tested in MMR-deficient tumors identified by PCR, regardless of origin. The FDA later granted accelerated approval for pembrolizumab to treat MSI or MMR-deficient solid tumors, including endometrial cancer (88–91). Although MSI or POLE-mutated status is commonly associated with favorable responses to immunotherapy, some endometrial cancers without these specific alterations have also shown responses (90–92). Ongoing clinical trials in MSS endometrial cancers aim to enhance the efficacy of immune checkpoint inhibitors by combining them with various agents, such as upfront chemotherapy, poly (ADP-ribose) polymerase (PARP) inhibitors, or antiangiogenic drugs. Additionally, the investigation of combination therapies with other immunotherapeutic agents and radiation therapy is of interest (93). Notable ongoing studies include the double-blind, placebo-controlled phase 3 AtTEnd trial (ClinicalTrials.gov identifier NCT03603184) that randomizes patients with metastatic or inoperable uterine carcinoma or carcinosarcoma to receive carboplatin and paclitaxel plus placebo or carboplatin, paclitaxel, and atezolizumab with placebo continued until disease progression (122).

Research is in the early preclinical stage on using natural killer T (NKT) cells to treat glioblastoma multiforme (GBM). Studies show NKT cells can kill GBM cell lines and reduce tumor burden in GBM xenograft mouse models (107, 123, 124). When NKT cells are administered along with α-GalCer (KRN7000), a synthetic glycosphingolipid, the survival of mice with intracranial tumors is enhanced. Type I NKT cells have been shown to exhibit killing activity against CD1d-positive GBM cell lines or patient-derived GBM cells after expansion with IL-2 and α-GalCer. The production of IFN-γ, TNF-α, granzyme B, and IL-4 is significantly increased. In an orthotopic GBM model, co-injecting human type I NKT cells with α-GalCer into tumor-bearing mice with CD1d-positive U251 cells significantly extends survival. However, in an intracranial injection model, type I NKT cells do not inhibit the growth of CD1d-negative U87 cells. This suggests that human-type I NKT cells specifically target CD1d-expressing GBM cells (108, 109).

With the FDA approval of pembrolizumab, immunotherapy has developed as a prominent treatment approach for triple-negative breast cancer (TNBC) (125). Although atezolizumab initially showed promise, its approval for advanced TNBC was withdrawn due to the lack of benefit observed in the IMpassion131 trial (110, 111). Further analysis of this trial is crucial to evaluate potential confounders, such as tumor-infiltrating lymphocytes (TILs), and determine if they were imbalanced between treatment arms, as observed in the NeoTRIPaPDL1 trial (112). In the curative setting, several studies have demonstrated a pathological complete response (pCR) benefit with the addition of immunotherapy to neoadjuvant chemotherapy regimens for early-stage TNBC (113). KEYNOTE-522 trial, pembrolizumab is now approved as part of neoadjuvant treatment and as a single-agent adjuvant treatment for high-risk early-stage TNBC (114). Ongoing trials such as IMpassion030 (NCT03498716) will provide further confirmation if this benefit extends to patients receiving solely adjuvant chemotherapy, who might potentially have a lower volume of immunogenic tumor antigens (115). Additionally, the value of adjuvant immunotherapy alone in patients with residual disease after neoadjuvant chemotherapy would be addressed by the SWOG 1418 trial (NCT02954874) (116).

There are increasing preclinical and clinical reports to evaluate various combinations of ICIs and cell-based therapies such as CAR-T cells and TILs (117, 118, 126–141). A promising evaluation of CHECKMATE-9ER is reported that combined nivolumab (Opdivo) and ipilimumab (Yervoy) in renal cell carcinoma (142). Notable ICIs for combinatorial anticancer therapy trials such as KEYNOTE-671 to evaluate pembrolizumab (Keytruda) + chemotherapy in triple-negative breast cancer (143–147). Also, JULIET is under evaluation as tisagenlecleucel (Kymriah) to treat relapsed/refractory diffuse large B-cell lymphoma. Another clinical trial on JCAR017 (lisocabtagene maraleucel) is reported in relapsed/refractory B-cell lymphomas (144). Clinical trials of IMPOWER150 are reported on the combined effects of atezolizumab (Tecentriq), carboplatin, and paclitaxel in non-squamous non-small cell lung cancer (146).

Besides ICIs in cancer immunotherapies, evaluation of CAR-T therapy as ZUMA-2 is presented to test the effects of axicabtagene ciloleucel (Yescarta) in refractory/relapsed follicular lymphoma (148). The combination of nivolumab, ipilimumab, and radiation therapy for glioblastoma is being explored. Additionally, an overview of clinical trials on cancer immunotherapy combined with other treatments is provided (142–152) (**Table 3**).

**Table 3:** A summary on clinical trials of cancer immunotherapies in combinatorial drug treatments for cancer patients.

Trial Name	Trial Phase/Clinical Trails Number	Target Indication	Therapeutic Approach	Key Outcomes/Summary	References
KEYNOTE-189	Phase 3 NCT02578680	NSCLC	ICI (pembrolizumab + chemo)	ORR: 48%, PFS: 9.0 mo, OS: 22.4 mo	(143, 147)
JULIET	Phase 2 NCT02445248	DLBCL	CAR-T (tisagenlecleucel)	ORR: 53%, DCR: 80%	(144)
CheckMate 067	Phase 3 NCT01844505	melanoma	ICI (nivolumab + ipilimumab)	ORR: 57%, PFS: 11.5 mo, OS: 58%	(142)
IMpower150	Phase 3 NCT02366143	NSCLC	ICI (atezolizumab + carboplatin + paclitaxel)	ABCP significantly improved OS compared to BCP, including in patients with EGFR/ALK mutations and liver metastases (ORR: 45%, PFS: 8.3 mo, OS: 19.4 mo)	(146, 153)
ZUMA-2	Phase 2 NCT02601313	follicular lymphoma	CAR-T (axicabtagene ciloleucel)	Single infusion of KTE-X19 led to durable responses in patients who had failed previous therapies, including BTK inhibitors (ORR: 95%, DCR: 100%)	(148, 154)
CHECKMATE-9ER	Phase 2 NCT03141177	Advanced renal cell carcinoma	Nivolumab + cabozantinib vs. sunitinib	Combination therapy demonstrated significant improvements in OS, and response rates with manageable safety profile (Median PFS: 16.6 months vs. 8.3 months; ORR: 55.7% vs. 27.1%)	(145, 151, 155)
TRANSCEND NHL 001	Phase 2 NCT02631044	NHL	CAR-T (lisocabtagene maraleucel)	ORR: 74%, DCR: 93%	(152)
KEYNOTE-522	Phase 2 NCT03036488	Triple-negative breast cancer	Pembrolizumab + chemotherapy vs. placebo + chemotherapy (neoadjuvant followed by adjuvant)	Addition of pembrolizumab to neoadjuvant chemotherapy significantly increased pCR rates and improved event-free survival in early TNBC (pCR rate: 64.8% (pembrolizumab) vs. 51.2% (placebo); 3-year EFS: 84.5% vs. 76.8%)	(142, 149, 150)

NSCLC, Non-Small Cell Lung Cancer; mCRC, Metastatic Colorectal Cancer; RCC, Renal Cell Carcinoma; NHL, Non-Hodgkin Lymphoma; AML, Acute Myeloid Leukemia; ICI, Immune Checkpoint Inhibitor; CAR-T, Chimeric Antigen Receptor T-cell therapy; mAb, Monoclonal antibody; OV, Oncolytic virus; ORR, Overall Response Rate; PFS, Progression-Free Survival; OS, Overall Survival; DCR, Disease Control Rate.

In recent, significant strides have been made to develop nucleic acid vaccines including various forms of DNA and RNA vaccines as a new form of arsenal in cancer immunotherapy (94–101, 156–158). The progressive intent behind the development of these cancer vaccines could be attributed to precision, high efficacy, and fewer side effects notable clinically approved drugs such as Cavatak (CF33) as DNA vaccines and mRNA-4157, BNT111 as RNA vaccines (97–101, 106, 157, 158). However, the effectiveness of DNA and mRNA cancer vaccines is limited by the evolving complexity and heterogeneity of the tumor immune microenvironment at both intra-tumoral and inter-tumoral levels. A summary table presents various nucleic acid vaccines for cancer immunotherapy, including their mechanisms and outcomes (97–101, 106, 157, 158) (**Table 4**).

**Table 4:** Summary on nucleic acid vaccines for cancer immunotherapies, with mechanisms and outcomes.

Vaccine Type	Approved Drug		Target Antigen	Cancer Type	Outcome	References
DNA vaccine	Cavatak (CF33)		MUC1, EGFR	Various	Enhanced anti-tumor immunity	(97)
RNA vaccine	mRNA-4157		PD-L1	Melanoma, NSCLC	Increased tumor-infiltrating lymphocytes	(98)
mRNA vaccine	BNT111		MUC1, CEA	Breast, lung, colorectal	Induced antigen-specific T-cells	(99)
Electroporated DNA vaccine	Intuvax (hTERT)		hTERT	Prostate cancer	Improved overall survival	(100)
Personalized neoantigen RNA vaccine	mRNA-5671		Mutated tumor antigens	Melanoma, lung cancer	Durable clinical responses	(101)
DNA vaccine	Vigil (p53)		p53	Breast, lung, colorectal	Enhanced anti-tumor immunity	(157)
RNA-Lipoplex vaccine	BI 1361849 (RNActive)		MUC1	Lung, pancreatic cancer	Increased tumor-specific T-cells	(158)
mRNA vaccine	CV8102		VEGFR2	Renal cell carcinoma	Reduced tumor growth	(106)

### 5.1 Microbiome and immunotherapy

In terms of innate immunity, the immune system actively shapes the GM composition from an early age, and the GM, in turn, influences the immune system’s development (102–104, 159–164). Key interactions between the gut microbiome (GM) and the immune system influence the development of a balanced GM and proper immune function from birth. Disruptions in GM composition, such as antibiotic use, can contribute to immune-related disorders later in life, including asthma and inflammatory bowel disease (104, 105, 165–168). Dysbiosis has the potential to influence both tumor development and the failure of ICI-based therapies overall. A balanced and diverse gut microbiome can activate the immune system to combat cancer and facilitate a robust response to anti-cancer immunotherapies, particularly ICIs (169–181). Several studies have established a correlation between gut microbiota (GM) composition and immunotherapy efficacy (102–105, 162–181).

Clinical data showed that tumors in antibiotic-treated or germ-free mice did not respond to anti-CTLA-4 immunotherapy. The anti-cancer effect of anti-CTLA-4 relied on Bacteroides species, particularly Bacteroides fragilis (160). Additionally, non-responder mice to anti-PD-L1 therapy showed improved responses when given feces from responder mice or orally administered Bifidobacterium. Another clinical finding pointed out that a higher proportion of Bacteroidetes was associated with reduced colitis risk in melanoma (MM) patients treated with immune checkpoint inhibitors (ICIs) (161, 180).

Frankel et al. (163) identified GM signatures associated with ICI efficacy in MM patients using metagenomic and metabolomic profiling. Responders had enriched *Bacteroides caccae, Faecalibacterium prausnitzii, Bacteroides thetaiotamicron, Holdemania filiformis*, and *Dorea formicogenerans*, with anacardic acid as a consistently enriched metabolite. Chaput et al. (164) found that MM patients with Bacteroides-driven baseline microbiota had longer progression-free survival (PFS) than those with Faecalibacterium-driven microbiota.

Matson et al. (102) identified enrichment of *Bifidobacterium longum, Collinsella aerofaciens*, and *Enterococcus faecium* in responders. Gopalakrishnan et al. (103) observed higher alpha diversity, Ruminococcaceae abundance, and enriched anabolic pathways in responders. Tanoue et al. (166) isolated an 11-strain bacterial consortium that enhanced CD8+ T-cell levels and MHC-I expression in dendritic cells (DCs). Xu et al. (167) showed *Prevotella* spp. and *Akkermansia* spp. can affect glycerol-lipid metabolism and anti-PD-1 efficacy. Mager et al. (168) found that *Bifidobacterium pseudolongum* produces inosine, which activates anti-tumor CD8+ T cells via the A2A receptor. Si W et al. (169) and Shi Y et al. showed that *Lactobacillus rhamnosus* GG and Bifidobacterium spp. enhance immunotherapy through the cGAS/STING pathway in dendritic cells. A summary of cancer immunotherapy and gut microbiome combinations, supported by preclinical and clinical evidence, is presented (168–181) (**Table 5**).

**Table 5:** Summary on the combined use of cancer immunotherapies and microbiotas for combinatorial therapies with preclinical and clinical evidences.

Immunotherapy	Microbiota-Based Approach	Mechanism	Preclinical/Clinical Evidence	Potential Benefits	References
Checkpoint Inhibitors	Fecal Microbiota Transplantation (FMT)	Enhance immune response, reduce immune suppression	Preclinical: improved tumor control, increased TILs	Enhanced efficacy, reduced toxicity	(175, 176)
CAR-T Cell Therapy	Microbiome modulation via dietary fiber	Improve CAR-T cell expansion, persistence	Preclinical: enhanced CAR-T cell efficacy	Increased treatment success	(177)
Cancer Vaccines	Probiotics (e.g., Lactobacillus)	Enhance antigen presentation, stimulate immune response	Clinical: improved vaccine efficacy in melanoma	Enhanced immune response	(178)
Adoptive T-Cell Therapy	Microbiota-derived metabolites (e.g., SCFAs)	Enhance T-cell function, reduce immune suppression	Preclinical: improved T-cell expansion, tumor control	Improved treatment outcomes	(179)
Oncolytic Viruses	Bifidobacterium-based therapy	Enhance virus-mediated tumor lysis, stimulate immune response	Preclinical: improved tumor control, increased survival	Enhanced antitumor efficacy	(180)
Immune Checkpoint Blockade	Akkermansia muciniphila-based therapy	Enhance immune response, reduce immune suppression	Clinical: improved treatment outcomes in melanoma	Enhanced efficacy, reduced toxicity	(181)

## 6 Challenges and future directions

Despite its success, cancer immunotherapy faces challenges that limit its effectiveness and widespread use. Cancer cells adapt by evading immune detection, downregulating target antigens, altering antigen presentation, and creating immunosuppressive microenvironments. Understanding these strategies is a key to improving treatments. Another major challenge is selecting the right patients for therapy. Not all patients respond equally to immunotherapy, and currently, we struggle to predict who will benefit most. The complex interplay between a patient’s immune system, their unique tumor characteristics, and even their gut microbiome make it difficult to design regimes and create combinations of medical, radiation and surgical oncology interventions. This heterogeneity makes identifying reliable predictive biomarkers challenging. When we activate the immune system against cancer, we sometimes trigger immune responses against healthy tissues as well. These irAEs can affect virtually any organ system and may force treatment interruptions or discontinuation. Finding the balance between maintaining therapeutic efficacy while minimizing these side effects remains a delicate challenge.

The high cost of immunotherapies, especially ICIs and CAR-T cell therapy, creates access disparities and strains healthcare systems. Addressing this economic barrier is crucial. Combining immunotherapy with other treatments like chemotherapy or radiation shows promise for better outcomes. However, identifying the optimal combination strategies and determining the optimal sequencing of therapies is complex. Comprehensive studies are needed to elucidate the most effective combinations and treatment sequences to maximize therapeutic synergies and minimize toxicity. SDDSs achieve this by co-delivering multiple therapeutic agents to tumor cells or immunosuppressive cells, thereby increasing drug concentration at the desired site and improving efficacy. They are used to reverse the immunosuppressive microenvironment created by tumors. Furthermore, SDDSs modulate the interferon signaling pathway and enhance the effectiveness of cell therapies. Understanding the microbiome at strain-level along with species-level in gut and TME can open up a new level of personalized medicine. Having that extra resolution is crucial if we are to understand what is happening in the human body and the interplay between cancer treatment and the microbiome. Being able to test the specific mechanisms of this relationship between specific strains and response is the next horizon in this research, and one that could benefit human health in a multitude of ways (94). Rare cancers are challenging to study and treat. While immunotherapy can be highly effective, its outcomes can be unpredictable. Research shows that the microbiome influences responses to combination immunotherapy, while monotherapy yields different results. This highlights the need to consider the microbiome when developing treatments. Additionally, live biotherapeutic products could provide beneficial bacteria to enhance immunotherapy, improving patient responses.

## 7 Conclusion

Immunotherapy has transformed the field of oncology and given patients fresh hope, revolutionizing the treatment of cancer. In several cancer types, it has demonstrated notable success by enabling the immune system to target cancer cells. To improve the outcomes of cancer immunotherapies in combinatorial treatments, a cohesive approach could involve developing strategies to address tumor cell resistance. There may be need for accessible and reliable data on predictive biomarkers that may be helpful for the selection of the right cancer immunotherapies for the right cancer patients.

The use of AI and machine learning can be adopted to decide the optimal dose and treatment modalities of combinatorial therapies involving various cancer immunotherapies.

For personalized medicines, concerted and comprehensive efforts are needed at the level of microbiome and outcome of cancer immunotherapies. Also, the expanded domain of cancer immunotherapies may be converged for rare cancers and, the influence of circadian rhythm on the success and failure of cancer patients.

In the future, oncolytic viruses, vaccines based on neoantigens, live biotherapeutics, immune engineering methodologies, and combinatorial avenues with engineered microbiomes can be explored as new forms of cancer therapeutics. Achieving this requires AI-driven predictive tools to optimize the type, dose, and timing of immunotherapies, alone or in combination.

An approach incorporating predictive biomarkers, AI-driven treatment personalization, and microbiome-based strategies will be instrumental in refining cancer immunotherapies. Biomarkers will help identify patients who will benefit the most, AI will optimize prognosis treatment selection, dosage, and response prediction, while microbiome-based interventions could enhance immunotherapy efficacy. Cancer immunotherapy is not just a promising frontier—it is a transformative force reshaping oncology. With strategic integration of advanced technologies, it holds the potential to shift cancer from a life-threatening disease to a manageable condition. To improve cancer treatment, CAR-T cells, mRNA, and DNA vaccines should be prioritized for greater efficacy, lower toxicity, personalized care, better accessibility, and long-term monitoring of side effects.
